# Description of the mitochondrial genomes of Sichuan *Tetrastes sewerzowi* (Galliformes: Tetraonidae) and phylogenetic relationship

**DOI:** 10.1038/s41598-024-77604-2

**Published:** 2024-11-05

**Authors:** Xuequn Luo, Hang Zhou, Ao Li, Zhaoxia Huang, Wenping Zhang, Shizhang Du, Donglai Hua

**Affiliations:** 1 College of Resources and Environmental Engineering, Mianyang Normal University, Mianyang, 621000 China; 2 College of Life Science and Biotechnology, Minshan Biodiversity Monitoring and Analysis Laboratory of Giant Panda National Park, Mianyang Normal University, Mianyang, 621000 China; 3 Mianyang Management Sub-Bureau of Giant Panda National Park, Mianyang, 621000 China

**Keywords:** *Tetrastes sewerzowi*, Mitochondrial Genomes, Phylogeny, Wanglang, DNA sequencing, Sequence annotation, Mitochondrial genome

## Abstract

The Chinese Grouse (*Tetrastes sewerzowi)*, a rare endemic bird in China, belongs to the Galliformes order and Tetraonidae family. Despite extensive research, subspecies classification remains inadequate, especially in Pingwu, Sichuan. This study sequenced the mitochondrial genome of *T. sewerzowi* in Wanglang Nature Reserve, Sichuan, analyzed its structural features, and reconstructed phylogenetic relationships within Tetraonidae. The results indicate that the high conservation in mitochondrial genome structure and sequence between the T. sewerzowi from Wanglang, Sichuan and Lianhuashan, Gansu indicates that they may belong to the same subspecies. Furthermore, we observed significant differences in the length of the mitochondrial control region between *T. sewerzowi* and *Tetrastes bonasia*. Based on the phylogenetic tree constructed using a comprehensive mitochondrial dataset, we propose the reclassification of the Hazel Grouse genus into two independent genera: *Tetrastes* and *Bonasa*. This study is the first to sequence the mitochondrial genome of *T. sewerzowi* from Sichuan and compare it with populations from Gansu, providing important insights into the distribution pattern of *T. sewerzowi* subspecies and facilitating the formulation of effective conservation strategies.

## Introduction

The Chinese Grouse (*Tetrastes sewerzowi)*, belongs to the Aves class, Galliformes order, Tetraonidae family. As a rare bird endemic to China, it is primarily distributed in the high-altitude forests of Gansu, Qinghai, and Sichuan provinces. It is a national-level protected animal, on the same level as the Giant Panda^1,2^, and was listed as a Near Threatened (NT) species by the International Union for Conservation of Nature (IUCN) in 1988. Despite the relevant departments having taken numerous positive measures to protect this species, including enhancing public awareness, cracking down on illegal trapping, and protecting its habitat, its endangered status has not yet been fundamentally altered. There is an urgent need for research and conservation efforts regarding this species.

Currently, research on the Chinese Grouse focuses mainly on its physiological and ecological behaviors, as well as phylogenetic relationships. The distribution of subspecies of the Chinese Grouse has not been studied in detail. It is known that the Chinese Grouse has differentiated into two subspecies: the nominate subspecies and the Sichuan subspecies. but some scholars are controversial about the distribution and classification of its subspecies. For instance, according to Zhao^3^ et al*.* in “A Checklist on Chinese Birds. Volume 1. Non-Passeriformes”, Sichuan Wanglang *T. sewerzowi* colony belongs to the Sichuan subspecies, while the colony at Lianhuashan, Gansu, is the nominate subspecies. However, in “Fauna Sinica. Volume 4. Aves. Galliformes”^4^, both the Wanglang *T. sewerzowi* and the population in Gansu’s Lotus Mountain are named nominate subspecies. Currently, there is only one complete mitochondrial genome of the Chinese Grouse from the Lianhuashan Nature Reserve, Gansu Province (KJ997914.1), available in GenBank^5^. The question of whether the Chinese Grouse from Lianhuashan, Gansu Province, and Wanglang, Sichuan Province, represents a subspecies of *T. sewerzowi* requires further genetic data for clarification.

Furthermore, although the phylogenetic relationships of the Tetraonidae family have been studied extensively, the phyloge- netic relationships among its internal lineages remain controversial. For instance, in taxonomic listings, the genera *Bonasa* and *Tetrastes* consistently appear adjacent to each other. Morphologically, Short et al*.*^6^ has argued that *Tetrastes* should be merged into *Bonasa* based on features such as plumage and mating behavior. On the molecular level, Dimcheff et al*.*^7^ employed the KH method^8^ to evaluate alternative tree topologies for Tetraonidae using a combined dataset of 12S and ND2 genes, revealing that *Bonasa umbellus*, *Tetrastes bonasia* / *T. sewerzowi* may form a monophyletic group, consistent with the morphological findings. However, Vittorio et al*.*^9^ utilizing CR and ND2 gene sequences and the TN93 nucleotide substitution model (Tamura-Nei,93) , found that the genetic distance between *B. umbellus* and *T. bonasia / T. sewerzowi* was significantly greater than the average genetic distance within the genera *Falcipennis*, *Lagopus*, and *Tetrao*, suggesting that maintaining *Bonasa* and *Tetrastes* as separate genera is appropriate. Consequently, further evidence is required to conclusively determine whether *Tetrastes* should be merged into *Bonasa*.

The mitochondrial genome, with its simple, highly conserved structure and matrilineal inheritance, is an important tool for studying the origin and evolution of species^10^. It plays an important role in the evolutionary classification and conservation of species. Take the endemic Chinese giant salamander as an example. Due to the lack of obvious external trait differences, current conservation measures in China are based on ‘the Chinese giant salamander is a species’, and are marketed for captive breeding and stocking, and do not differentiate between populations in different places. However, several analyses, including mitochondrial genetics, have shown that the Chinese giant salamander is not a single species^11^. If conservation strategies are not adjusted in time, some species may not only be unprotected, but also accelerate extinction. The successful application of mitochondrial genome technology in the conservation of the Chinese giant salamander shows that mitochondrial genome research can help reveal genetic differences and species delimitations within species, and provide examples for the conservation of other endangered species.

Therefore, the aim of this study was to sequence the mitochondrial genome and analyse the mitochondrial genome structure of the Chinese Grouse inhabiting Wanglang Nature Reserve in Sichuan, China, with the aim of providing a reference for the distribution of subspecies of this species and the development of conservation strategies. At the same time, we reconstructed the phylogenetic relationships of the Tetraonidae family by combining the mitochondrial data of Tetraonidae family birds available in public databases, and provided molecular biological information for the taxonomic study of Tetraonidae family birds.

## Results

**Fig. 1:**
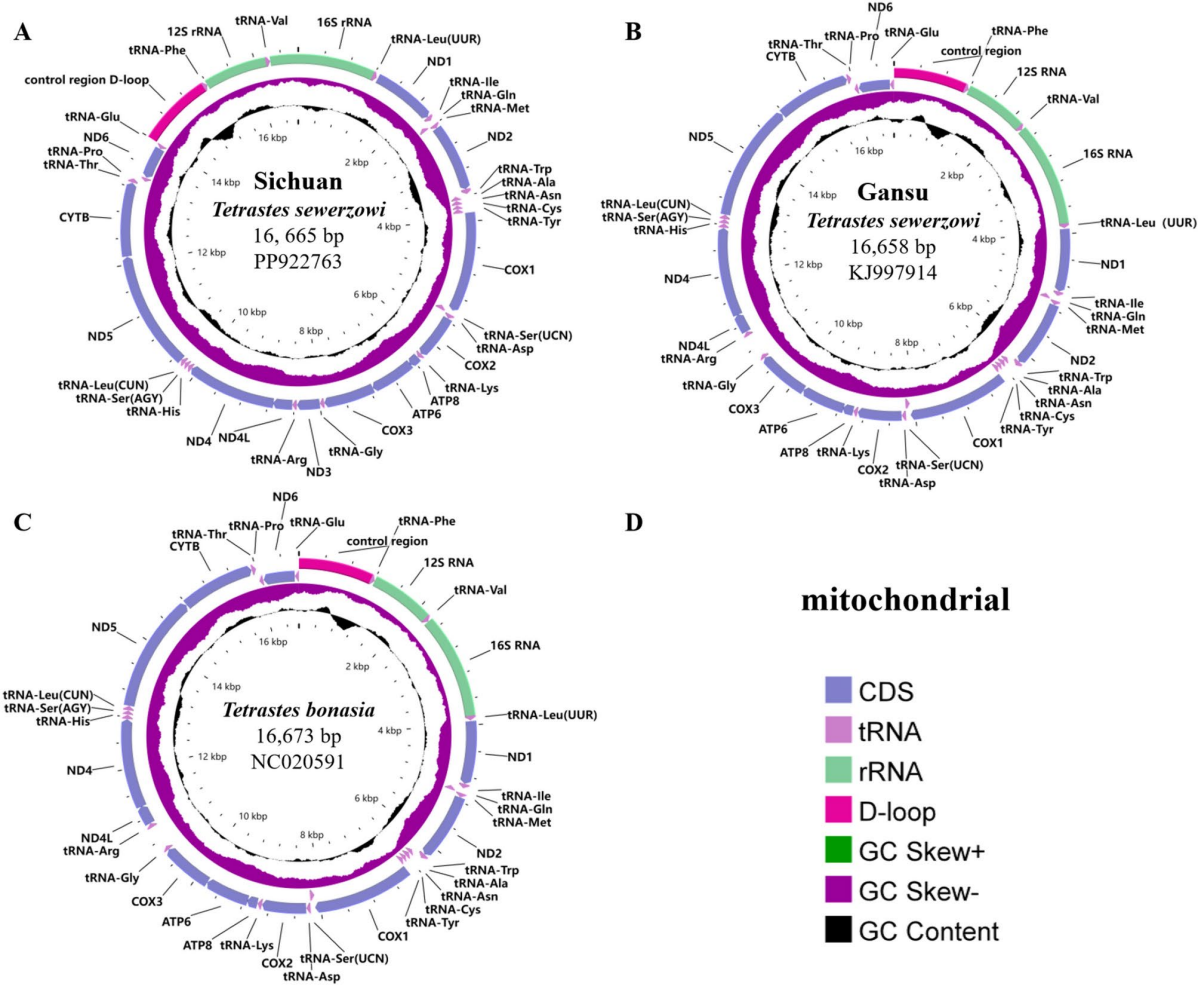
Mitochondrial genome characteristics of two species of *Tetrastes*. (**A**) Mitochondrial map of Sichuan *T. sewerzowi*; (**B**) Mitochondrial map of Gansu *T. sewerzowi*; (**C**) Mitochondrial map of *T. bonasia*; The legend is depicted in (**D**).

The mitochondrial genome of the newly sequenced Chinese Grouse is a closed-loop molecule, measuring 16,665 base pairs (bp.) in length, which is comparable to the mitochondrial genomes of other birds belonging to the Tetraonidae family. This genome comprises 13 protein-coding genes (PCGs), 22 tRNA genes, two rRNA genes, and a control region (CR), and the gene composition and order were consistent with that of *T. bonasia*^9^ (NC020591) and Gansu *T. sewerzowi*^5^ (KJ997914) (Fig. 1) . Comparison revealed that the mitochondrial genomes of the Chinese Grouse and *T. bonasia* had the longest overlap region (

10 bp) between ATP8 and ATP6, and the longest spacer region between ND1 and tRNA-Leu (UUR) . Among them, the spacer was 16 bp in Sichuan *T. sewerzowi* and shorter at 9 bp in *T. bonasia* (Table 1). Furthermore, the mitochondrial genomes of the Chinese Grouse from Sichuan and Gansu provinces exhibit identical AT base composition, AT-skew, and GC-skew (Table 2) .

**Table 1:** Annotation of the complete mitogenome of the two *Tetrastes* species.

Gene	Start Codon	Stop Codon	Anti-Codon	strand	Intergenic Nucleotides (IGN)
D-loop				H	0
tRNA-Phe			GAA	H	−1 (b)/0 (a, c)
12S rRNA				H	0
tRNA-Val			TAC	H	0
16S rRNA				H	0 (a)/−1 (b, c)
tRNA-Leu (UUR)			TAA	H	16 (a)/10 (b)/9 (c)
ND1	ATG	TAA		H	0
tRNA-Ile			GAT	H	7
tRNA-Gln			TTG	L	−1
tRNA-Met			CAT	H	0
ND2	ATG	T**		H	0
tRNA-Trp			TCA	H	2
tRNA-Ala			TGC	L	2 (a, b)/3 (c)
tRNA-Asn			GTT	L	2
tRNA-Cys			GCA	L	−1
tRNA-Tyr			GTA	L	1
COX1	GTG	AGG		H	−9
tRNA-Ser (UCN)			TGA	L	1 (a)/2 (b, c)
tRNA-Asp			GTC	H	1
COX2	ATG	TAA		H	1
tRNA-Lys			TTT	H	1
ATP8	ATG	TAA		H	−10
ATP6	ATG	TAA		H	−1
COX3	ATG	T**		H	0 (a, b)/1 (c)
tRNA-Gly			TCC	H	0
ND3	ATG	TAA		H	1
tRNA-Arg			TCG	H	0
ND4L	ATG	TAA		H	−7
ND4	ATG	T**		H	0
tRNA-His			GTG	H	1 (a, c)/0 (b)
tRNA-Ser (AGY)			GCT	H	2 (a)/0 (b)/1 (c)
tRNA-Leu (CUN)			TAG	H	0
ND5	ATG	TAA		H	4
CYTB	ATG	TAA		H	2
tRNA-Thr			TGT	H	0
tRNA-Pro			TGG	L	6
ND6	ATG	TAG		L	1
tRNA-Glu			TTC	H	0

H and L refer to the heavy and light strands; CR = control region. ‘/’ indicates type of intergenic nucleotides in *Tetrastes* species: (**a**) Sichuan *T. sewerzowi*; (**b**) Gansu *T. sewerzowi*; (**c**) *T. bonasia.*

**Table 2:** Nucleotide composition of the mitochondrial genome of the two *Tetrastes* species.

Nucleotide compos	Sichuan *T. sewerzowi*	Gansu *T. sewerzowi*	*T. bonasia*
*Whole Genome*			
A + T (%)	55.70	55.70	55.90
AT-Skew	0.08	0.08	0.08
GC-Skew	−0.40	−0.40	−0.39
*PCGS*			
A + T (%)	55.20	55.20	55.50
AT-Skew	0.02	0.02	0.02
GC-Skew	−0.42	−0.42	−0.41
*tRNAs*			
A + T (%)	57.40	57.40	57.50
AT-Skew	0.06	0.06	0.06
GC-Skew	−0.03	−0.03	−0.03
*16S rRNA*			
A + T (%)	57.00	57.00	56.10
AT-Skew	0.20	0.20	0.21
GC-Skew	−0.20	−0.19	−0.19
*12S rRNA*			
A + T (%)	52.90	52.80	54.00
AT-Skew	0.24	0.24	0.22
GC-Skew	−0.20	−0.20	−0.19
*Control region*			
A + T (%)	59.10	59.40	59.40
AT-Skew	0.13	0.11	0.13
GC-Skew	−0.30	−0.31	−0.30

### Protein-encoding genes and codon usage preferences

The A + T content of protein-coding genes (PCGs) within the mitochondrial genome of *T. sewerzowi* from Sichuan and Gansu was 55.20%, marginally lower than that observed in the PCGs of *T. bonasia*’s mitochondrial genome, which exhibited an A + T content of 55.50% (Table 2). Across the 13 PCGs from these two species, the initiation and termination codons were largely conserved, with the exception of COX1, which employed GTG as its start codon, whereas the remaining 12 PCGs strictly adhered to the canonical start codon ATG (Table 1). The mitochondrial genomes of these two species encompass four stop codons (TAA, AGG, TAG, and an incomplete codon T), with the ND4, ND2, and COX3 genes utilizing the incomplete stop codon T.

**Fig. 2:**
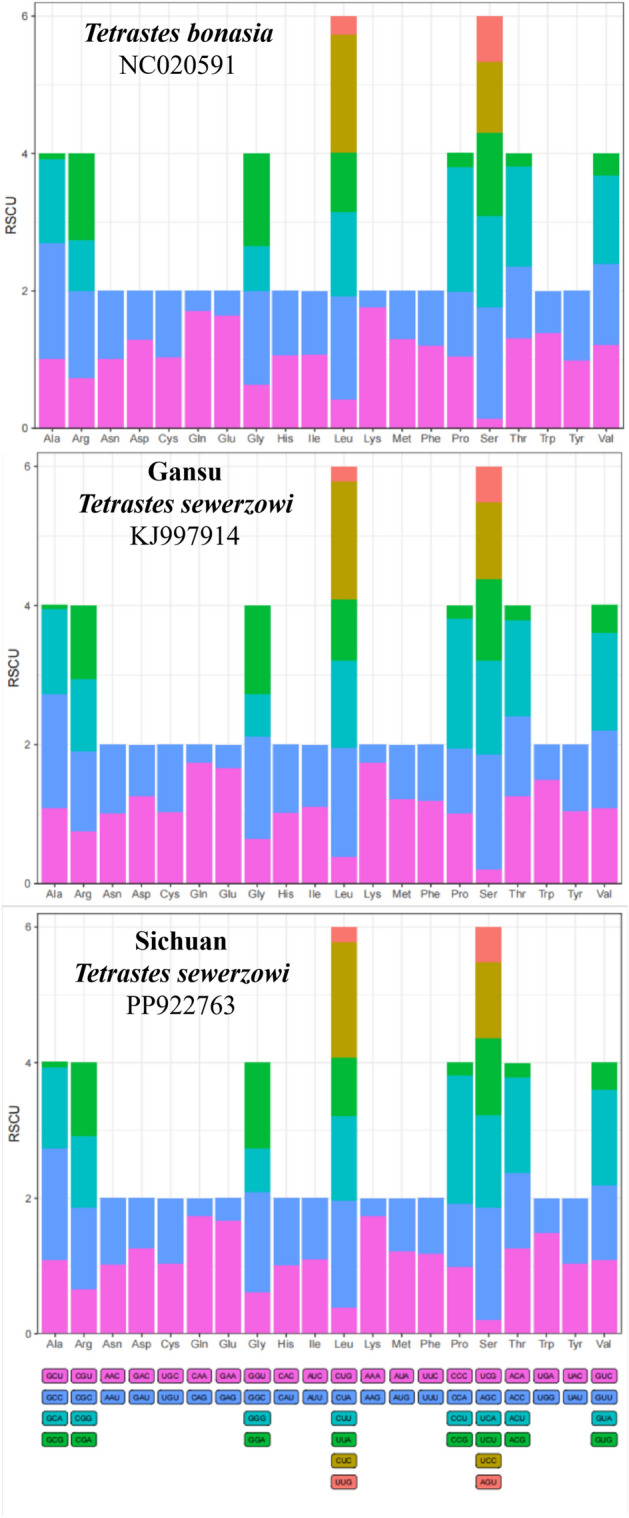
Relative synonymous codon usage (RSCU) for protein-coding genes of the three mitochondrial genomes. Codon families are provided on the x-axis.

An analysis of the relative synonymous codon usage (RSCU) of the 13 PCGs in these three mitochondrial genomes revealed that CCU (Pro), CUC (Leu), and AUC (Ile) were the most prevalent codons across all three mitochondrial genomes (Fig. 2). Notably, the highest RSCU values for the mitochondrial-encoded genes of *T. sewerzowi* from both Sichuan and Gansu were CCU (Pro), AAA (Lys), and CAA (Gln), respectively, the two local populations, Gansu *T. sewerzowi* and Sichuan *T. sewerzowi*, are consistent but distinct from that of *T. bonasia*, where the top three RSCU values were CCU (Pro), CAA (Gln) , and AAA (Lys). Furthermore, the analyses emphasized the high A + T base content of the PCGs in these three genomes (Table 2) and the dominance of codons terminating in A or T (Table 3).

**Table 3:** Percentage (%) of GC base content at codons 1, 2 and 3 in protein coding genes (PCGs).

GC (%) base content	Sichuan *T. sewerzowi*	Gansu *T. sewerzowi*	*T. bonasia*
1st letter GC (%)	47.17	47.62	47.67
2nd letter GC (%)	44.56	44.85	44.75
3rd letter GC (%)	41.74	41.93	41.90

### rRNA and tRNA genes

The three mitochondrial genomes have 22 typical tRNAs ranging from 65 to 78 bp in size. Prediction of tRNA secondary structures using the software RNAscan-SE revealed that the tRNA-Ser (AGY) of the two species lacked the dihydrouracil loop, while the other tRNAs could all be folded into typical tRNA cloverleaf structures. Additionally, U-U, U-G, and C–C, C-A, and A-A mismatched base pairs were identified in the stems of 22 different tRNAs, with amino acid acceptor arms showing more mismatches compared to the other arms. The 16S rRNA and 12S rRNA were situated between tRNA-Val and tRNA-Leu and between tRNA-Phe and tRNA-Val, respectively (Fig. 1).

The 16S rRNA of Sichuan *T. sewerzowi* was located between tRNA-Val and tRNA-Leu, with a length of 1,610 bp, and the 12S rRNA was located between tRNA-tRNA-Phe and tRNA-Val, with a full length of 968 bp, which was consistent with the other two genomes (Fig. 1). Upon base composition analysis, it was found that the 16S rRNA and 12S rRNA of the three mitochondrial genomes had a greater A + T content than G + C content, and all of them were A- and C-skewed (Table 2).

### Control region

The control region in these two species is positioned between the tRNA-Phe and tRNA-Glu genes (Fig. 1). The sequence length of the control region in Sichuan *T. sewerzowi* genome was 1127 bp, identical to that of Gansu *T. sewerzowi*, and 14 bp shorter than that of *T. bonasia* (1141 bp) . The A + T content of the mitochondrial genome control region of Sichuan *T. sewerzowi* ranged from 59.1%, with an AT-skew of 0.13 and a GC-skew of -0.30. For Gansu *T. sewerzowi*, the A + T content of the mitochondrial genome control region ranged from 59.4%, with an AT-skew of 0.11 and a GC-skew of -0.31, while *T. bonasia* displayed an A + T content of 59.4%, an AT-skew of 0.13, and a GC-skew of -0.30, revealing minor differences among the two species (Table.2) .

**Fig. 3:**
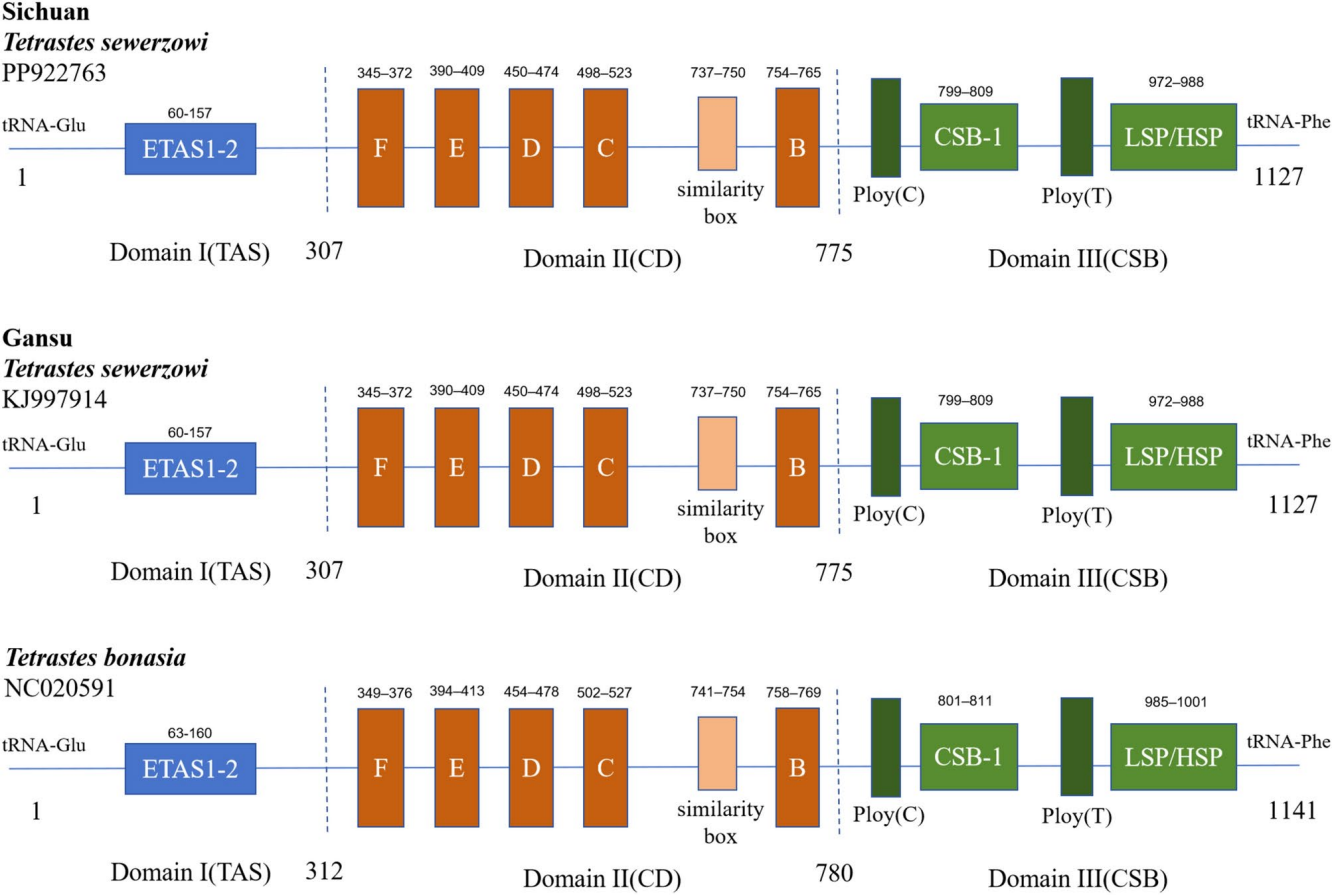
Structure prediction of mitochondrial control area in two species of *Tetrastes*. The extended termination-related sequences are represented by blue boxes, the sequence blocks in the central conserved region are represented by orange boxes, and the conserved sequence region is represented by green boxes.

In this study, we analyzed the control regions of the two species with reference to the Sitta^12^ and Gansu *T. sewerzowi*^5^, and the predicted structures are shown in Fig. 3. The mitochondrial control region sequences can be categorized into extended termination-associated sequence (ETAS) , central conserved domains (CD) and conserved sequence blocks (CSB) . The ETAS includes ETAS1 and ETAS2, while the CD can be sequentially divided into CSB-F, CSB-E, CSB-D, CSB-C and CSB-B blocks. The CSB includes the CSB-1 sequence and the light chain/heavy chain promoter (LSP/HSP) (Fig. 3).

### Phylogenetic analyses

**Fig. 4:**
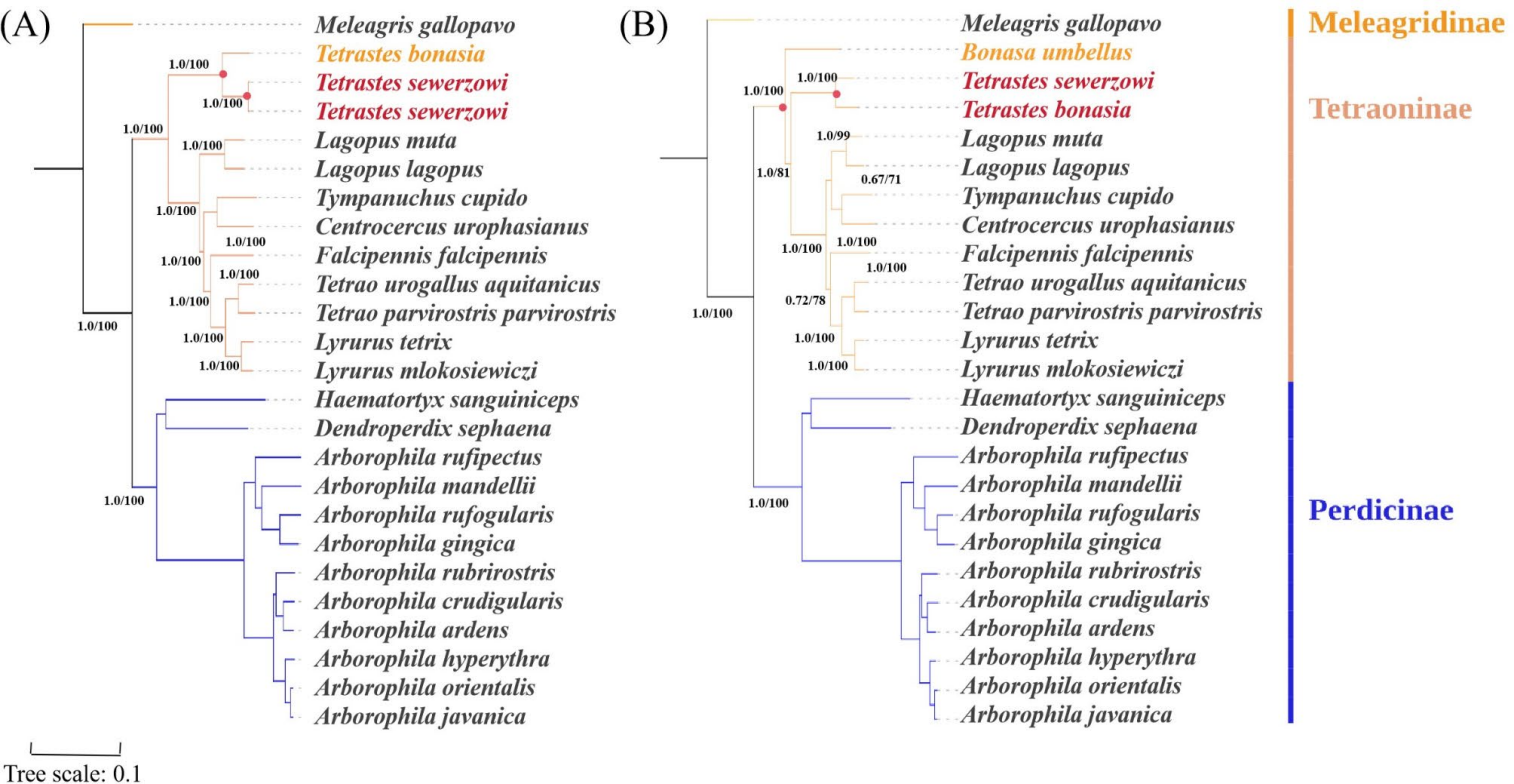
Phylogenetic trees of mitochondrial gene sequences, constructed using Bayesian inference and maximum likelihood analysis. The values displayed at the nodes represent posterior probabilities (PP) from Bayesian inference and bootstrap support values (BP) from maximum likelihood analysis. (**A**) Phylogenetic tree based on the full mitochondrial genome; (**B**) Phylogenetic tree constructed solely from the combined sequences of the CYTB, ND2, and 12S genes within the mitochondrial genome. *Note* Phylogenetic Genus Species Delimitation Label Reference NCBI Database).

To further elucidate the phylogenetic relationships within the Tetraonidae family, the present study conducted comprehensive phylogenetic analyses utilizing the complete mitochondrial genome of Tetraonidae species alongside sequences of pivotal genes, namely ND2, CYTB, and 12S rRNA (Table 4) . These analyses were performed using Maximum Likelihood (ML) and Bayesian Inference (BI) methods, with *Meleagris gallopavo* serving as the outgroup reference. The outcomes of these analyses revealed a high degree of congruence in the topologies of the phylogenetic trees derived from both ML and BI analyses, based on the aforementioned datasets. The corresponding Bayesian posterior probabilities (PP) and ML bootstrap support values (BP) are presented in Fig. 4.

**Table 4:** List of species used for phylogenetic analyses in this study.

No	ID	Species	Family	Genus	Length	A + T%
1	NC010195	*Meleagris gallopavo*	Meleagridinae	*Meleagris*	16,719	56.5
2	KJ716444	*Arborophila ardens*	Perdicinae	*Arborophila*	16,730	54.9
3	MN868242	*Arborophila crudigularis*	Perdicinae	*Arborophila*	16,733	55.3
4	FJ752425	*Arborophila gingica*	Perdicinae	*Arborophila*	16,728	54.1
5	MN868244	*Arborophila hyperythra*	Perdicinae	*Arborophila*	16,721	54.7
6	MN868245	*Arborophila javanica*	Perdicinae	*Arborophila*	16,723	55.1
7	MN868246	*Arborophila mandellii*	Perdicinae	*Arborophila*	16,724	55.3
8	MN868247	*Arborophila orientalis*	Perdicinae	*Arborophila*	16,727	55.1
9	MN868248	*Arborophila rubrirostris*	Perdicinae	*Arborophila*	16,729	54.5
10	NC012453	*Arborophila rufipectus*	Perdicinae	*Arborophila*	16,728	54.4
11	FJ752424	*Arborophila rufogularis*	Perdicinae	*Arborophila*	16,726	54.7
12	MW574367	*Dendroperdix sephaena*	Perdicinae	*Dendroperdix*	16,716	54.3
13	KY411591	*Haematortyx sanguiniceps*	Perdicinae	*Haematortyx*	16,692	53.0
14	AF222541	*Bonasa umbellus* (ND)	Tetraoninae	*Bonasa*	1041	57.8
15	KC785605	*Bonasa umbellus* (12S)	Tetraoninae	*Bonasa*	967	53.4
16	KX534430	*Bonasa umbellus* (CYTB)	Tetraoninae	*Bonasa*	1002	52.7
17	MW574357	*Centrocercus urophasianus*	Tetraoninae	*Centrocercus*	16,697	55.8
18	MW574370	*Falcipennis falcipennis*	Tetraoninae	*Falcipennis*	16,688	56.5
19	NC035568	*Lagopus lagopus*	Tetraoninae	*Lagopus*	16,677	55.9
20	KX609785	*Lagopus muta*	Tetraoninae	*Lagopus*	16,687	56.0
21	MW574375	*Lyrurus mlokosiewiczi*	Tetraoninae	*Lyrurus*	16,693	56.1
22	NC024554	*Lyrurus tetrix*	Tetraoninae	*Lyrurus*	16,677	56.2
23	MK820678	*Tetrao parvirostris parvirostris*	Tetraoninae	*Tetrao*	16,693	55.9
24	MG583885	*Tetrao urogallus aquitanicus*	Tetraoninae	*Tetrao*	16,683	55.9
25	NC020591	*Tetrastes bonasia*	Tetraoninae	*Tetrastes*	16,673	55.9
26	KJ997914	*Tetrastes sewerzowi*	Tetraoninae	*Tetrastes*	16,658	55.7
27	PP922763	*Tetrastes sewerzowi* (this study)	Tetraoninae	*Tetrastes*	16,665	55.6
28	MW574394	*Tympanuchus cupido*	Tetraoninae	*Tympanuchus*	16,697	56.0

The mitochondrial genomes in the above table are complete except for *B. umbellus*. Species nomenclature reference to NCBI database.

A thorough examination of the complete mitochondrial genome data revealed that the majority of species clustered tightly in accordance with their genus-level classification. Notably, Sichuan *T. sewerzowi* and Gansu *T. sewerzowi* exhibited a robust clustering pattern on a shared branch, with exceptionally strong node support (PP = 1.00, BP = 100) (Fig. 4A). In phylogenetic analyses of combined ND2, CYTB, and 12S gene sequences (Fig. 4B), *B. umbellus* did not form the same branch as *T. bonasia*/*T. sewerzowi*, and branch node support was high. These observations provide important insights to further elucidate the evolutionary dynamics among grouse species in the Tetraoninae.

## Discussion

### Mitochondrial structural characterization

The newly sequenced mitochondrial genome of Sichuan *T. sewerzowi*, as presented in this study, spans a total length of 16,665 bp, consisting of a double-stranded circular molecule that encompasses 37 typical genes and a control region. Its length and structure exhibit similarities to those of the mitochondrial genomes of Tetraonidae species published by the National Center for Biotechnology Information (NCBI)^5^. Comparative analyses reveal that the mitochondrial genomes of Sichuan *T. sewerzowi* and Gansu *T. sewerzowi* are highly conserved in terms of their structure and sequence order, both displaying comparable A + T content and AT / GC-skew.

Genetic diversity analysis reflects the ability of species to adapt to diverse environments and their evolutionary potential^13^, and it is an indispensable prerequisite for developing effective conservation strategies^14^. As a close relative of the Chinese Grouse, *T. bonasia* exhibits many morphological similarities^5^. Currently, *T. bonasia* has mitigated the risk of endangerment through breeding and other methods, whereas the Chinese Grouse has remained near-threatened since 1988. Comparing the mitochondria of *T. bonasia*^15^ and the Chinese Grouse to observe differences, although it does not directly increase the population of the Chinese Grouse, it can provide an important scientific basis and strategic support for its conservation.

It was found that there are many structural similarities between the mitochondrial genomes of *T. bonasia* and the Chinese Grouse. For instance, most protein-coding genes (PCGs) have complete stop codons, whereas ND4, ND2, and COX3 have incomplete stop codons (T**) . For incomplete termination codons, the missing nucleotide may be the result of post- transcriptional polyadenylation, which can generate functional termination codons through the mechanism of transcriptional cleavage and polyadenylation of polycistronic transcripts^12^. This is common in animal mitochondrial genomes. Among the tRNA genes, all are able to fold into the typical tRNA cloverleaf structure, except for trnS-AGY, which lacks a dihydrouridine loop. The lengths of 12S rRNA and 16S rRNA are consistent in both species.

It is important to note that, although the mitochondrial control region (CR) shares elements that regulate transcription and replication of the mitochondrial genome and exhibits significant AT-skew between the two species, it differs in sequence length. The control region of Chinese Grouse is 14 bp shorter than that of *T. bonasia*, and it is the region with the most significant difference in length in the mitochondrial genome. The mitochondrial control region is known for its rapid rate of variation and significant length variation^16^. It not only participates in and regulates the replication and transcription of mtDNA but also serves as a hub for the exchange of information between the mitochondria and the nuclear genome. Any damage or mutation in this region may cause structural and functional changes in the mtDNA and may even affect the function of the entire cell^17^. This, in turn, may impact the survival and reproduction of the species in different environments.

### Phylogenetic analysis

Due to the lack of complete *B. umbellus* genome sequences in the NCBI database, this study constructed a phylogenetic tree using a dataset of CYTB, ND2, and 12S mitochondrial genes. The tree showed that *T. sewerzowi* populations from Gansu and Sichuan clustered with high support (PP = 1.00; BP = 100), suggesting conserved mitochondrial gene structure. Populations in Lianhua Mountain, Gansu, and Wanglang, Sichuan, may belong to the same subspecies. In contrast, *B. umbellus* did not cluster with *T. sewerzowi* or *B. bonasia*. Based on these findings, we propose splitting the hazel grouse genus into *Tetrastes* and *Bonasa*.

### Protection measures and prospects

The *T. sewerzowi*, a unique grouse species endemic to China, primarily inhabits the alpine coniferous forests of Gansu, Qinghai, southwestern Sichuan, Yunnan, and eastern Tibet, particularly in the central and western high-altitude regions. This species is undergoing rapid population decline due to intensified habitat fragmentation, combined with hunting activities, predator threats, and parasitic infestations, necessitating urgent scientific research and conservation measures^18^. Current conservation strategies encompass habitat preservation, dynamic monitoring, control of human disturbances, and ecological restoration, such as implementing in-situ conservation to curb habitat destruction by human activities, constructing ecological corridors to enhance connectivity among forest patches^19^, and utilizing radio telemetry and satellite remote sensing technologies to deeply explore the physiological and ecological characteristics of *T. sewerzowi*^20^, thereby precisely grasping its population dynamics and ecological needs. These initiatives are crucial for ensuring the stable reproduction of *T. sewerzowi* populations and biodiversity conservation.

However, biodiversity conservation encompasses three levels: genetic, species, and ecosystem diversity. Current research primarily focuses on *T. sewerzowi* populations in Gansu, with insufficient attention paid to those in Sichuan, especially in Wanglang. This research imbalance may weaken genetic diversity conservation efforts, threatening the overall survival capacity of the species. This study focuses on the mitochondrial genetic structure of *T. sewerzowi* populations in Wanglang, Sichuan, revealing conservation in mitochondrial structure between populations in Lianhuashan, Gansu, and Wanglang, suggesting that similar conservation strategies can be adopted for both regions. However, it is noteworthy that *T. sewerzowi* populations in Songpan, Maerkang, Kangding, and other areas in Sichuan have not been adequately studied and may face unique ecological and genetic challenges, necessitating more detailed and targeted conservation measures.

To address this research gap, it is recommended to intensify exploration of the genetic diversity, ecological habits, and distribution range of *T. sewerzowi* populations in Sichuan to obtain more comprehensive data support. Based on these research findings, tailored conservation strategies for *T. sewerzowi* populations in Sichuan should be developed, encompassing habitat preservation, population monitoring, and ecological restoration. Simultaneously, strengthening cross-regional cooperation mechanisms is essential to jointly advance the overall conservation of *T. sewerzowi* and safeguard the future of this rare species.

## Conclusion

In this study, the mitochondrial genome of Sichuan *T. sewerzowi* was sequenced for the first time and compared with related species in terms of mitochondrial structure and number of bases. It was shown that the mitochondrial genomes of *T. sewerzowi* from Lianhuashan, Gansu and Wanglang, Sichuan were conserved in terms of genome structure, gene order and base composition; and the length of the mitochondrial control region of *T. sewerzowi* differed significantly from that of *T. bonasia*. Based on the phylogenetic tree constructed from the combined mitochondrial dataset, it was concluded that the Hazel Grouse genus should be divided into the genera *Tetrastes* and *Bonasa*.

## Methods

### DNA extraction and sequencing

In this study, faecal samples from suspected Chinese Grouse were collected in May 2023 within Wanglang Nature Reserve, Sichuan Province, China^1^. One sample positively identified as Chinese Grouse underwent whole-genome sequencing. The protocol entailed the following steps: faecal DNA was extracted for host species identification using the QIAamp® Fast DNA Stool Mini Kit (Qiagen, Germany) , adhering to the kit’s instruction manual, and subsequently dissolved in 50 L of TE buffer provided with the kit. Subsequently, primers (Forward: 5’-ATGAAGGGATGTTCTACTGGGTTG-3’; Reverse: 5’-AACATCTCCGCATGATGAA-3’)^21,22^ were employed to amplify the avian CYTB sequence, targeting a fragment length of 1200 bp, as described in previous studies. PCR amplification was conducted in a 40 L reaction mixture comprising 20 L of 2 × T8 High-Fidelity Master Mix, 2 L of each primer at 10 M, 2 L of DNA template, and 14 L of ddH2O. The PCR conditions were as follows: initial denaturation at 98 °C for 2 min, followed by 37 cycles of denaturation at 98 °C for 10 s, annealing at 55 °C for 15 s, and extension at 72 °C for 15 s, with a final extension at 72 °C for 5 min and a hold at 4 °C.

The PCR products were sequenced by the Chengdu Branch of Beijing Genomics Institute (BGI) Biotechnology Co., Ltd. The resulting sequences were submitted to NCBI for BLASTn analysis. In the comparison outcomes, the species displaying 99% or higher homology and the highest degree of homology, with clear species information, were selected as the reference for identification. Ultimately, high-throughput sequencing was conducted on the NovaSeq 6000 platform (Illumina, USA) for samples that passed quality control, utilizing a PE150 sequencing read length. Each read underwent bipartite sequencing, yielding approximately 60 Gb of raw data upon completion of the sequencing process.

### Mitochondrial genome assembly and annotation

The raw data underwent mitochondrial whole-genome assembly using GetOrganelle (v1.7.7.0)^23^. The mitochondrial group was annotated with Mitos2 (http://mitos2.bioinf.uni-leipzig.de) using the vertebrate mitochondrial genetic code^24^. The assembly was independent of the reference genome and was further refined with Geneious Prime (2024.0.4) (https://www.geneious.com) through alignment with homologous gene sequences from other Tetraonidae species. The entire mitochondrial genome of Sichuan *T. sewerzowi* has been preserved in GenBank (PP922763) . The mitochondrial genome was then mapped using PROKSEE (https://proksee.ca/)^25^, first upload your sequence to the website. Then, click on the “Features” button under the “Tool” section and upload your annotation file. After that, proceed to adjust the colors, sizes, and arrangement of the map as desired.

### Bioinformatics analysis

PCG, tRNA, CR, and rRNA genes were statistically analyzed for base composition and offset using Geneious Prime (2024.0.4) (https://www.geneious.com) ; tRNAscan-SE Search Server (http://lowelab.ucsc.edu/tRNAscan-SE/) was used to locate and estimate the secondary structure of tRNA genes^26^. Sequence comparison of coding genes was performed using MAFFT (v7.505)^27^. Codon preferences were calculated using EMBOSS explorer (https://www.bioinformatics.nl/emboss-explorer/). Relative synonymous codon usage (RSCU) of protein coding genes was calculated using MEGA (7.0)^28^. The formulas AT-skew = (A—T) / (A + T) and GC-skew = (G—C) / (G + C) were used for component skew analysis.

### Phylogenetic tree analysis

The mitochondrial gene sequences of Tetraonidae species and *M.gallopavo* species were downloaded from NCBI (refer to Table 4). In conjunction with the newly sequenced mitochondrial genomes, we constructed two phylogenetic trees: one based on the complete mitochondrial genome and another on a combination of ND2, CYTB, and 12S gene data. Throughout the entire process, we utilized the PhyloSuite (v1.2.3) software package^29,30^, with the specific steps outlined as follows:

Firstly, redundant sequences were filtered. Then, in the “Extract” window, we selected the appropriate extraction mode and codon table matching the data. Subsequently, MAFFT (v7.505)^27^ was employed for multiple sequence alignment, MACSE (v2.06)^31^ was used to optimize the alignment of protein-coding gene (PCG) sequences, and Gblocks (0.91b)^32^ was applied to trim the PCG sequences. Additionally, trimAl (v1.2rev57)^33^ was utilized to trim RNA sequences. A tandem PCGs + 12S RNA dataset was generated, excluding the third codon position. ModelFinder (v2.3.2)^34^ was then employed to select the optimal partitioning strategy and evolutionary model for the PCGs + RNA dataset. Finally, the phylogenetic tree was reconstructed using the maximum likelihood (ML) method with IQ-TREE^35^ software. Bayesian inference (BI) phylogenetic tree reconstruction was performed using MrBayes^36^ and the tandem dataset. A tandem PCGs + 12S RNA dataset was generated, excluding the third codon position. ModelFinder (version 2.3.2)^34^ was then applied to identify the optimal partitioning strategy and evolutionary model for this PCGs + RNA dataset, with the Bayesian Information Criterion (BIC) and corrected Akaike Information Criterion (AICc) serving as the respective criteria. Subsequently, maximum likelihood (ML) and Bayesian inference (BI) phylogenetic trees were reconstructed using IQ-TREE^35^ and MrBayes^36^ software, respectively. Finally, iTOL^37^ was employed to visually enhance the phylogenetic tree structure.

## Data Availability

Sequence data that support the findings of this study have been deposited in the Genebank with the primarv accession code PP922763.
